# Yi-Zhi-Fang-Dai Formula Exerts Neuroprotective Effects Against Pyroptosis and Blood–Brain Barrier–Glymphatic Dysfunctions to Prevent Amyloid-Beta Acute Accumulation After Cerebral Ischemia and Reperfusion in Rats

**DOI:** 10.3389/fphar.2021.791059

**Published:** 2021-12-15

**Authors:** Zhongkuan Lyu, Qiyue Li, Zhonghai Yu, Yuanjin Chan, Lei Fu, Yaming Li, Chunyan Zhang

**Affiliations:** ^1^ Geriatrics Department of Chinese Medicine, Huadong Hospital Affiliated to Fudan University, Shanghai, China; ^2^ Department of Traditional Chinese Medicine, Shanghai Jiao Tong University Affiliated Sixth People’s Hospital, Shanghai, China; ^3^ Shanghai Key Laboratory of Clinical Geriatric Medicine, Huadong Hospital Affiliated to Fudan University, Shanghai, China; ^4^ International Medical Center of Traditional Chinese Medicine, Haikou Hospital of Traditional Chinese Medicine, Haikou, China

**Keywords:** Yi-Zhi-Fang-Dai formula, cerebral ischemia and reperfusion injury, dementia, neuroinflammation, pyroptosis, blood–brain barrier–glymphatic system, aquaporin-4, amyloid-beta peptide

## Abstract

**Background:** The dysfunctional blood–brain barrier (BBB)–glymphatic system is responsible for triggering intracerebral amyloid-beta peptide (Aβ) accumulation and acts as the key link between ischemic stroke and dementia dominated by Alzheimer’s disease (AD). Recently, pyroptosis in cerebral ischemia and reperfusion (I/R) injury is demonstrated as a considerable mechanism causing BBB–glymphatic dysfunctions and Aβ acute accumulation in the brain. Targeting glial pyroptosis to protect BBB–glymphatic functions after cerebral I/R could offer a new viewpoint to prevent Aβ accumulation and poststroke dementia. Yi-Zhi-Fang-Dai formula (YZFDF) is an herbal prescription used to cure dementia with multiple effects of regulating inflammatory responses and protecting the BBB against toxic Aβ-induced damage. Hence, YZFDF potentially possesses neuroprotective effects against cerebral I/R injury and the early pathology of poststroke dementia, which evokes our current study.

**Objectives:** The present study was designed to confirm the potential efficacy of YZFDF against cerebral I/R injury and explore the possible mechanism associated with alleviating Aβ acute accumulation.

**Methods:** The models of cerebral I/R injury in rats were built by the method of middle cerebral artery occlusion/reperfusion (MCAO/R). First, neurological function assessment and cerebral infarct measurement were used for confirming the efficacy of YZFDF on cerebral I/R injury, and the optimal dosage (YZFDF-H) was selected to conduct the experiments, which included Western blotting detections of pyroptosis, Aβ_1-42_ oligomers, and NeuN, immunofluorescence observations of glial pyroptosis, aquaporin-4 (AQP-4), and Aβ locations, brain water content measurement, SMI 71 (a specific marker for BBB)/AQP-4 immunohistochemistry, and Nissl staining to further evaluate BBB–glymphatic functions and neuronal damage.

**Results:** YZFDF obviously alleviated neurological deficits and cerebral infarct after cerebral I/R in rats. Furthermore, YZFDF could inactivate pyroptosis signaling via inhibiting caspase-1/11 activation and gasdermin D cleavage, ameliorate glial pyroptosis and neuroinflammation, protect against BBB collapse and AQP-4 depolarization, prevent Aβ acute accumulation and Aβ_1-42_ oligomers formation, and reduce neuronal damage and increase neurons survival after reperfusion.

**Conclusion:** Our study indicated that YZFDF could exert neuroprotective effects on cerebral I/R injury and prevent Aβ acute accumulation in the brain after cerebral I/R associated with inhibiting neuroinflammation-related pyroptosis and BBB–glymphatic dysfunctions.

## Introduction

Alzheimer’s disease (AD), a common neurodegenerative disease, comprises the major type of dementia and is causing a high socioeconomic impact with the advancement of world population aging (Alzheimer’s Association, 2021). It is known that amyloid-beta peptide (Aβ) accumulation acts as a core factor among the multifaceted etiology of AD, which is closely associated with the cerebrovascular dysfunctions, especially brain microcirculation disturbance (Bell and Zlokovic, 2009; Yamazaki and Kanekiyo, 2017; Jack et al., 2018; Hampel et al., 2021; Kim et al., 2021). Experimental and clinical research is emerging to indicate that cerebral ischemia and reperfusion (I/R) can trigger both acute and chronic accumulation of Aβ in the brain which exacerbates cerebral I/R injury and accounts for the occurrence of dementia induced by ischemic stroke (van Groen et al., 2005; Song et al., 2013; Liu et al., 2015; Martins et al., 2019). Thus, maintaining the clearance of Aβ after cerebral I/R could offer a new therapeutic approach to prevent poststroke cognitive impairment and development into dementia (Goulay et al., 2020).

The normal blood–brain barrier (BBB) is an essential condition for keeping the balance of intracerebral and extra-cerebral Aβ, and accordingly BBB breakdown has been demonstrated as an early biomarker prior to appearance of cognitive impairment (Nation et al., 2019; Hussain et al., 2021). In addition to the BBB, the glymphatic system is another considerable pathway for the clearance of Aβ in the brain (Tarasoff-Conway et al., 2015). Endfeet of astrocytes are the main components of both the BBB and glymphatic system which contribute to maintain the homeostasis of brain microenvironments, and aquaporin-4 (AQP-4) on astrocytic endfeet is a water channel protein with high polarization and essential for neurovascular coupling and glymphatic flow to facilitate the clearance of metabolites such as Aβ (Nakada et al., 2017). Thus, in cerebral I/R injury, the loss of AQP-4 polarization on astrocytic endfeet is considered as an important factor of BBB–glymphatic dysfunctions that are the vital pathological change causing the onset and development of dementia (Verheggen et al., 2018; Goulay et al., 2020).

Inflammation is inherent across the whole course of both ischemia and reperfusion stages, and accordingly neuroinflammation acts as the fundamental cause and meanwhile as the consequence of cerebral I/R injury (Liu et al., 2014; Du et al., 2021). Recently, pyroptosis, a pro-inflammatory cell death, has been demonstrated as a crucial pathological link and gasdermin D (GSDMD) as its key effector in cerebral I/R injury (Zhang et al., 2019). Canonical pyroptosis was deemed to rely on the activation of inflammasomes represented by nucleotide-binding oligomerization domain-like receptors pyrin domain-containing 3 (NLRP3)/apoptosis-associated speck-like protein containing a caspase activation and recruitment domain (ASC)/caspase-1 to cleave GSDMD, causing the secretion of pro-inflammatory mediators such as cleaved interleukin-1β (IL-1β) (Shi J. et al., 2015). However, in the noncanonical pyroptosis pathway, GSDMD is the direct substrate of caspase-11 (orthologous caspase-4/5 in humans), and the N-terminal fragment (GSDMD-N) from the cleavage of full length GSDMD (GSDMD-FL) is critical for the formation of membrane nanopores leading to cell death. Meanwhile, as the upstream signaling, GSDMD-N activates the NLRP3/ASC/caspase-1 pathway and then results in the maturation and secretion of cleaved IL-1β (Kayagaki et al., 2015; Yi, 2018; Matikainen et al., 2020). Our recent study (Lyu et al., 2021) indicated that caspase-11-mediated pyroptosis after cerebral I/R focuses on glial cells (microglia and astrocytes) and is a considerable factor aggravating BBB–glymphatic dysfunctions and Aβ accumulation.

As one hallmark of cerebral I/R injury, BBB breakdown in the ischemic period is exacerbated by reperfusion and followed by a no-reflow phenomenon of capillaries (Mohamed Mokhtarudin and Payne, 2015; Burrows et al., 2016; Huang et al., 2020). In addition to intracerebral Aβ retention caused by microcirculation disturbance, activated platelets contained in microthrombosis during the ischemic period and after I/R are demonstrated as the potential peripheral source of acute Aβ accumulation in blood vessels including capillaries and nearby brain tissues (Martins et al., 2019; Carbone et al., 2021). Aβ accumulation in the brain can raise the formation of a toxic Aβ-like Aβ_1-42_ oligomer, cause swelling in astrocytic endfeet, and also lead to dysregulation of capillaries by acting on pericytes, impairing energy supply for neurons (Merlini et al., 2011; Cavallucci et al., 2012; Nortley et al., 2019). Therefore, early protection of microcirculation and elimination of thromboinflammation in microcirculation after cerebral I/R are both crucial therapeutic strategies for the clearance of metabolites such as Aβ and the prevention of poststroke dementia.

According to traditional Chinese medicine (TCM) theories, blood stasis in brain collaterals with stagnancy of collateral-Qi in a deficiency condition is regarded as the basic pathogenesis of cerebral I/R injury (Wang B. et al., 2021), thus invigorating Qi and dredging brain collaterals are basic TCM therapeutic principles which consist of current therapeutic strategies emphasizing on microcirculation protection and removal of obstructions. Herbs have been widely used for thousands of years and are suitable for treating complex diseases such as ischemic stroke and dementia with the multicomponent and multitarget advantages (Yu et al., 2020; Singh et al., 2021). Yi-Zhi-Fang-Dai formula (YZFDF) is an experiential herbal prescription (Chan et al., 2020) commonly used to cure dementia cases by multiple efficacies of invigorating Qi, dredging brain collaterals, and promoting neurological function recovery. YZFDF is purely composed of herbal medicines with various bioactive ingredients, such as bilobalide, ginkgolide A, ginsenoside Rg1, cistanoside A, and α-asarone (Liu et al., 2016a). Our previous work showed that YZFDF and EGb761, the extracts from its main herb (*Ginkgo biloba* leaves), can inhibit microglial activation, regulate inflammatory responses, and protect the BBB against toxic Aβ-induced damage *in vitro* and *in vivo* (Wan WB. et al., 2014; Wan et al., 2016; Chan et al., 2020). Therefore, based on our previous studies, the present study was designed to confirm the potential therapeutic effects of YZFDF against cerebral I/R injury and further preliminarily explore the possible mechanism associated with alleviating Aβ acute accumulation by anti-neuroinflammation–related pyroptosis and BBB–glymphatic dysfunctions.

## Materials and Methods

### Components and Drug Powder Preparation of YZFDF

YZFDF comprises four herbs as shown in Table 1, including *Ginkgo Biloba* leaves, Ginseng, Cistanches Herba, and grassleaf sweetflag rhizome, which were purchased from Shanghai Hongqiao Pharmaceutical Co., Ltd. (Shanghai, China). These herbal medicines were identified by the TCM Preparation Room of Shanghai Geriatric Institute of Chinese Medicine, Shanghai University of Traditional Chinese Medicine. The main active ingredient analysis of YZFDF by the methods of high-performance liquid chromatography (HPLC) and mass spectrometry (MS), as well as the chemical structures of each ingredient were introduced in detail in our previous work (Liu et al., 2016a). The YZFDF drug powder was prepared as previously described (Liu et al., 2016a; Chan et al., 2020). In brief, four herbal medicines were subjected twice to extraction with 75% ethanol for 2 h. The herbal dregs of the extract solution were removed after filtering. Subsequently, the filtered liquid was concentrated using a rotary evaporator (BÜCHI Labortechnik AG, Flawil, Switzerland) and then dried to get a drug powder by the freeze-drying method. The YZFDF power was kept in an airtight container in the deep freezer (−36°C) for long-term storage, and the powder was made into a suspension liquid nearing usage and then stored at 4°C.

**TABLE 1:** Components of Yi-Zhi-Fang-Dai formula.

Latin name	English name	Chinese name	Part used	Ratio (%)
*Ginkgo biloba*	Ginkgo biloba leaves	Yinxingye	Dry leaves	30
*Panax ginseng* C. A. Meyer	Ginseng	Renshen	Root and rhizome	30
*Cistanche deserticola* Ma	Cistanches Herba	Roucongrong	Succulent stem	30
*Acorus tatarinowii*	Grassleaf sweetflag rhizome	Shichangpu	Rhizome	10

### Animals

All animal experiments in this study were approved by the Ethics Committee of Shanghai Jiao Tong University Affiliated Sixth People’s Hospital and performed in accordance with the relevant guidelines and regulations. Efforts were made as well to minimize animal suffering during the whole experiments. Specific pathogen-free (SPF) male Sprague–Dawley (SD) rats, weighing 200–230 g, were purchased from the Shanghai Laboratory Animal Research Center (Shanghai, China). The rats were housed in a SPF barrier environment under standard conditions at a controlled temperature (23 ± 1°C) on a 12:12 h light–dark cycle. Experimental operations were carried out after the acclimation of animals for several days with free access to food and water.

### Drug Administration and Experimental Design

The common human daily dosage of raw YZFDF herbs is 100 g/75 kg bodyweight. According to the formula (Lan et al., 2013) d_rat_ = d_human_ × 0.7/0.11, the common dosage of raw YZFDF herbs in rats should be 8.48 g/kg/day, and the corresponding drug powder dosage is 2.69 g/kg/day. The drug tolerance of a rat is generally higher than that of the human, and thus we selected 2.8, 5.6, and 11.2 g/kg/day as the low, medium, and high drug powder dosages of YZFDF in the present study, respectively. Accordingly, at the first stage of this study, forty rats were randomly divided into five groups: sham group (Sham), ischemia and reperfusion group (I/R), YZFDF low-dosage group (YZFDF-L), YZFDF medium-dosage group (YZFDF-M), and YZFDF high-dosage group (YZFDF-H). The rats in the YZFDF-treated groups were orally administered with the corresponding drug powder dosage of YZFDF (dissolved in distilled water), and the other rats were given the same volume of distilled water. Drug administration was performed twice a day at 9:00 and 16:00 for 3 days before the surgery and lasted 3 days after the surgery until animal sacrifice. According to the results of neurological function assessment and measurement of cerebral infarct area, the optimal dosage of YZFDF (YZFDF-H) was selected to conduct the following experiments.

**FIGURE 1:**
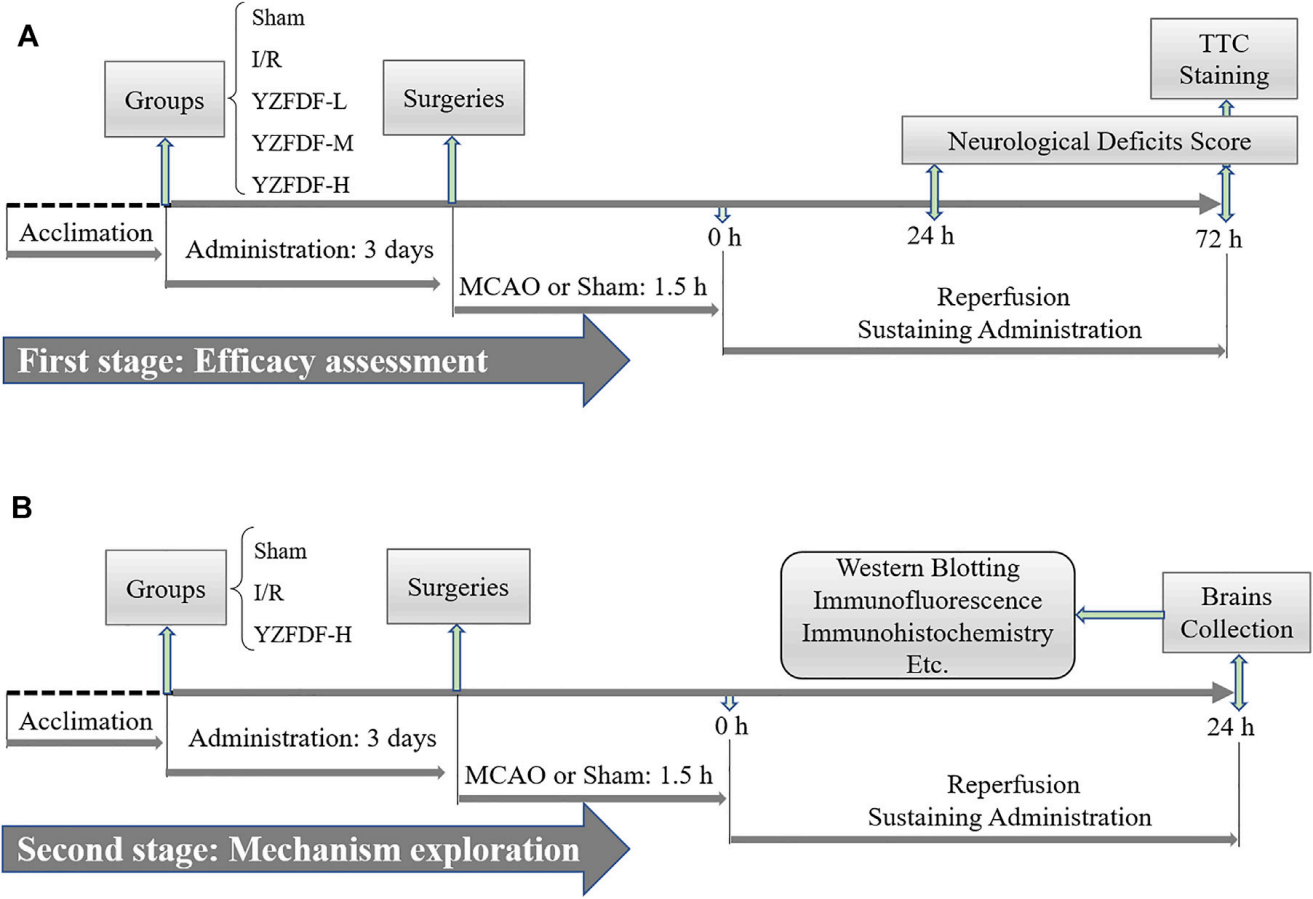
Schematic diagram of this study. **(A)** First stage aiming at the efficacy assessment of YZFDF against I/R injury. **(B)** Second stage of this study for further preliminarily exploring the possible mechanism.

At the second stage of this study, forty-five rats were randomly divided into three groups: sham group (Sham), ischemia and reperfusion group (I/R), and YZFDF high-dosage group (YZFDF-H). Drug administration was performed as previously mentioned for 3 days before the surgery and lasted until animal sacrifice for 24 h after reperfusion. The schematic diagram of this study is exhibited in Figure 1.

### Focal Cerebral I/R Injury Models

The models of focal cerebral I/R injury were built by the method of left middle cerebral artery occlusion/reperfusion (MCAO/R) as described in our previous studies (Yu et al., 2016; Yu et al., 2018). In brief, rats were anesthetized with pentobarbital sodium (0.5%, 1 ml/100 g). After the skin disinfection and incision, the left common carotid artery (CCA) was identified and exposed by separation from surrounding tissues. Subsequently, the external carotid artery (ECA) and internal carotid artery (ICA) were dissected and exposed carefully. At first, the ICA was occluded using a microvascular clip and the far end of the ECA was fastened, followed by the ECA being cut at 1 cm away from the bifurcation between the ECA and ICA. Then, a nylon monofilament (Beijing Sunbio Biotech, China) was inserted into the ICA from the incision of the ECA with the microvascular clip removed. At last, resistance could be felt when the rounded tip of the monofilament reached the origin of the middle cerebral artery (MCA) at the length of 18.5–19.5 mm from the bifurcation, and then the monofilament was fastened at ECA stump. After MCAO of 1.5 h, the monofilament was withdrawn to implement reperfusion. In the present study, the rats in I/R and YZFDF-treated groups were subjected to blinded MCAO/R surgeries, while rats in the Sham group only underwent the same operation with no insertion of the monofilament. During the whole course, the cardiovascular rate and rectal temperature of all rats were monitored and maintained.

### Neurological Function Assessment

Neurological examinations were performed after reperfusion. In order to exclude the interference of surgery failures, the rats subjected to MCAO/R with no detectable neurological deficits were eliminated. The neurological deficit scores of rats at 24 and 72 h after reperfusion in the present study were evaluated on a 5-point scale as previously described (Longa et al., 1989; Cai et al., 2016): 0 = no deficit; 1 = failure to extend right forepaw; 2 = circling to the right; 3 = falling to the right; 4 = no spontaneous walking with a depressed level of consciousness.

### Measurement of the Cerebral Infarct Area

The measurement of the cerebral infarct area by 2, 3, 5-triphenyl tetrazolium chloride (TTC; Nanjing Jiancheng Bioengineering Institute, Nanjing, Jiangsu, China) staining was carried out as previously described (Yu et al., 2016). In brief, at 72 h after reperfusion, the rats under deep anesthesia went through cardiac perfusion with 200 ml normal saline. Subsequently, their brains were taken out quickly and placed at −20°C for 20 min. Then, each brain was sliced into five coronal slices (2 mm thickness) from the rostral to the caudal on the frozen ice pack, and then the slices were stained with TTC solution away from light for 20 min at 37°C. As a result, the infarct brain tissue was stained to the white-colored area distinguished from the red-colored non-infarct area. After the fixation of stained brain slices with 4% paraformaldehyde for 24 h, the percentages of the cerebral infarct area were calculated by microscope image analysis software (Image-Pro Plus, United States) according to the following formula (Yu et al., 2016): [contralateral hemisphere area-(ipsilateral hemisphere area-infarct area)/2×contralateral hemisphere area] × 100%.

### Brain Water Content Measurement

The dry–wet weight method was used to measure the brain water content. In Brief, the rats were sacrificed under deep anesthesia, and their brains were quickly taken out. Then, ischemic and nonischemic cerebral hemispheres were separated, immediately weighed to obtain the wet weight (WW), and then placed in an oven at 60°C for 24 h to obtain the dry weight (DW). The brain water content was calculated with the following formula (Lan et al., 2013): 100% × (WW−DW)/WW.

### Western Blotting Analysis

After 24 h reperfusion, the rats were deeply anesthetized and went through cardiac perfusion. The brains were taken out, and the ischemic core and normal brain tissue were obviously visible to the naked eye. Then, the transition zone neighboring the ischemic core (ischemic penumbra) and the equivalent area under sham were quickly peeled off and stored at −80°C. Western blotting (WB) analysis was used to detect the expression levels of neuroinflammation-related pyroptosis signaling molecules, Aβ_1-42_ monomer/oligomers, and NeuN (a marker of neurons). In brief, the brain tissues from ischemic penumbra and the equivalent area under sham were prepared for protein samples followed by the concentration measurement. Subsequently, corresponding protein samples with equal amounts were separated by 10% sodium dodecyl sulfate-polyacrylamide gel electrophoresis and then electrotransferred onto the poly-vinylidnene fluoride membranes. Then, the membranes were blocked by 5% bovine serum albumin (BSA) at room temperature for 1 h and incubated at 4°C overnight with the following primary antibodies (Supplementary Table S1 for details): caspase-11 (Santa Cruz, United States), GSDMD (CST, United States), NLRP3 (ProteinTech, United States), ASC (Santa Cruz), caspase-1 (ProteinTech), IL-6 (Santa Cruz), IL-1β (Santa Cruz), ionized calcium-binding adapter molecule-1 (Iba-1) (Abcam, United Kingdom), Aβ_1-42_ (Abcam), NeuN (ProteinTech), and β-actin (CST). Then, the membranes were washed and incubated with the corresponding secondary antibodies (Signalway Antibody, United States) for 1 h at room temperature. Finally, the enhanced chemiluminescence kit (Millipore, United States) was used to develop WB bands, and the intensities of bands were analyzed with ImageJ software (National Institutes of Health, United States).

### Immunofluorescence

After deep anesthetization, the rats went through cardiac perfusion with 200 ml normal saline and then 4% paraformaldehyde. Subsequently, the brains were taken out and immersed in 4% paraformaldehyde for 24 h fixation and then prepared for paraffin slices. The procedure for immunofluorescence (IF) staining of proteins colocalization was as follows: after dewaxing and rehydration with gradient ethanol (100% ethanol for 5 min, 95% ethanol for 5 min, 80% ethanol for 5 min, 60% ethanol for 5 min, and H_2_O for 5 min), the slices further went through antigen retrieval and permeation by 0.3% triton-X 100 followed by blockage with 5% BSA. Subsequently, the slices were incubated with the first antibodies (Supplementary Table S1 for details) mixed with GSDMD/Iba-1, GSDMD/glial fibrillary acidic protein (GFAP), AQP-4/GFAP, and Aβ/GFAP overnight at 4°C followed by incubations with corresponding mixed secondary antibodies (Beyotime, China) for 1 h at room temperature. After DAPI staining, the laser scanning confocal microscope (Leica Wetzlar, Germany) was used for capturing fluorescent pictures in the same brain area with blinding. Three fields were randomly selected for the analysis of double-positive staining cell number or fluorescent density by ImageJ software.

### Immunohistochemistry

An endothelial barrier antigen (EBA, clone: SMI 71) is a specific marker for the BBB, and AQP-4 polarization loss is an important cause of BBB–glymphatic dysfunctions responsible for Aβ accumulation. Thus, we further made evaluations of BBB–glymphatic functions by immunohistochemistry (IHC) staining of SMI 71 and AQP-4. In brief, after dewaxing and rehydration, the slices were subjected in sequence to antigen retrieval, permeation, inactivation of the endogenous catalase, and then blockage by 5% BSA. Subsequently, the slices were incubated with the anti-rat BBB antibody (SMI 71) (BioLegend, United States) and AQP-4 antibody (Santa Cruz) overnight at 4°C (Supplementary Table S1 for details), followed by incubations with the corresponding secondary antibodies for 1 h at room temperature. Then, 3,3-diaminobenzidine tetrahydrochloride and hematoxylin were used for visualizing the slices. Finally, the light microscope was used for observing the slices and capturing the pictures in the same brain area with blinding. Three fields were randomly selected for the analysis of SMI 71 and AQP-4 staining density by ImageJ software.

### Nissl Staining

Nissl staining was used to evaluate neuronal damage as described in our previous study (Yu et al., 2016). In brief, after dewaxing and rehydration, the slices were stained with Nissl staining solution (Sangon Biotech, China) for 20 min at room temperature. Subsequently, the slices were rinsed in graded ethanol, transparentized by xylene, and coverslipped under Permount. Finally, the pictures in the same brain area were captured with blinding using the light microscope. Four fields were randomly selected for the analysis of damaged neurons.

### Statistical Analysis

All data were expressed as the mean ± standard deviation (SD) or standard error of the means (SEM). GraphPad Prism 8.0 (GraphPad Software Inc. United States) was used for statistical analysis. The differences among groups were analyzed by one-way ANOVA or unpaired Student’s t-test. A value of *p* < 0.05 was considered to be statistically significant.

## Results

### Yi-Zhi-Fang-Dai Formula Alleviated Neurological Deficits After Cerebral I/R in Rats

**FIGURE 2:**
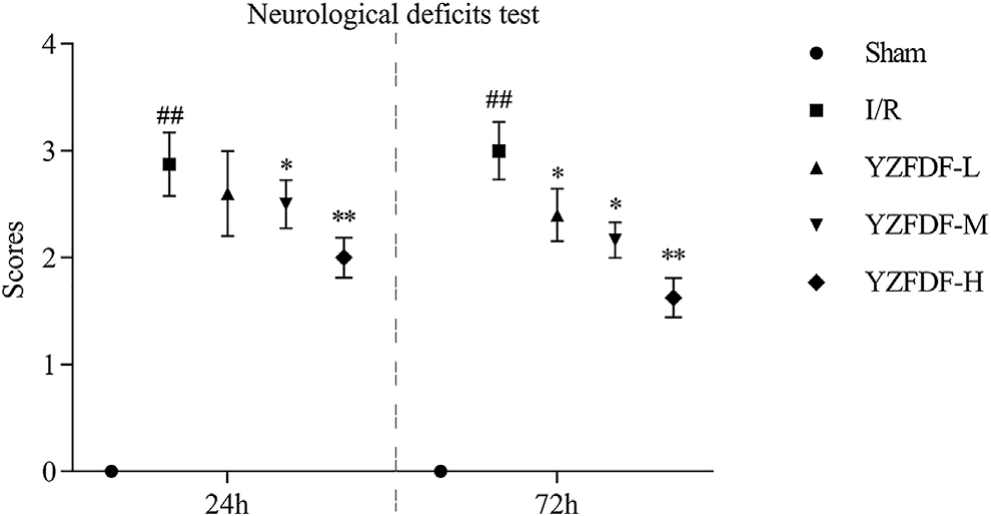
YZFDF alleviated neurological deficits at 24 and 72 h after cerebral I/R in rats, *n* = 5–8. ^##^ *p* < 0.01 vs. Sham group; ^*^ *p* < 0.05, ^**^ *p* < 0.01 vs. I/R group.

First, the effects of YZFDF on neurological deficits were assessed at 24 and 72 h after reperfusion in the present study. As exhibited in Figure 2, the rats in the Sham group had no performance of neurological deficits, and the rats in the I/R group showed obvious neurological deficits at both 24 and 72 h after reperfusion. However, compared with that of the I/R group, YZFDF-treated groups showed significantly low neurological deficit scores at 24 h (YZFDF-M group, *p* < 0.05; YZFDF-H group, *p* < 0.01) and 72 h (YZFDF-L and YZFDF-M groups, *p* < 0.05; YZFDF-H group, *p* < 0.01) after reperfusion. Thus, the result of neurological deficit assessment indicated that YZFDF could alleviate neurological deficits after cerebral I/R in a dosage- and time-dependent manner*.*

### Yi-Zhi-Fang-Dai Formula Reduced Cerebral Infarct After Cerebral I/R in Rats

**FIGURE 3:**
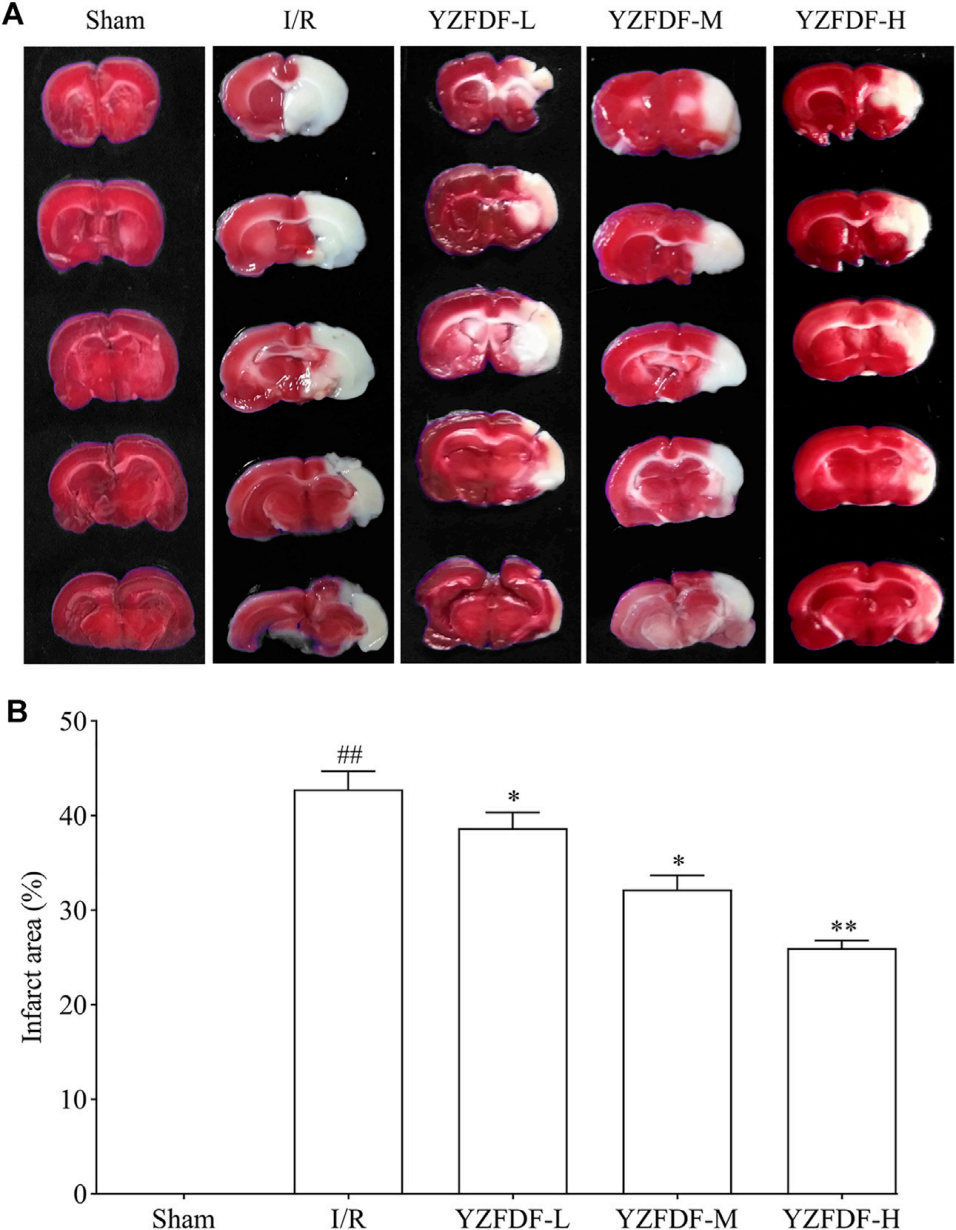
YZFDF reduced cerebral infarct at 72 h after cerebral I/R in rats. **(A)** Representative pictures of cerebral infarct by TTC staining in each group. The red-colored area represents normal cerebral tissue with non-infarct, and the white-colored area represents cerebral infarct. **(B)** Comparative analysis of the cerebral infarct area between groups, *n* = 5–8. ^*^ *p* < 0.05, ^**^ *p* < 0.01 vs. I/R group.

The effects of YZFDF on cerebral infarct were measured following neurological deficit assessment at 72 h after reperfusion. The result (Figures 3A,B) showed that the rats in the Sham group had no cerebral infarct, and the rats in the I/R group exhibited obvious cerebral infarct (white-colored area). However, compared with that of the I/R group, YZFDF-treated groups showed significantly little cerebral infarct area with the YZFDF-H group exerting optimal effects (YZFDF-L and YZFDF-M groups, *p* < 0.05; YZFDF-H group, *p* < 0.01), which was consistent with the result of neurological deficit assessment*.*

Based on the outcomes at the first stage of this study and our previous work, the following experiments selected the optimal dosage of YZFDF (YZFDF-H) to further explore the potential effects of YZFDF against the fundamental and crucial links in cerebral I/R injury represented by neuroinflammation-related pyroptosis, BBB–glymphatic dysfunctions, and Aβ acute accumulation and thus to preliminarily probe into its neuroprotective mechanism for preventing early pathological changes of poststroke dementia.

### Yi-Zhi-Fang-Dai Formula Alleviated Cerebral I/R–Induced Pyroptosis *via* Inhibiting the Activation of Caspase-11/1 and Cleavage of GSDMD

**FIGURE 4:**
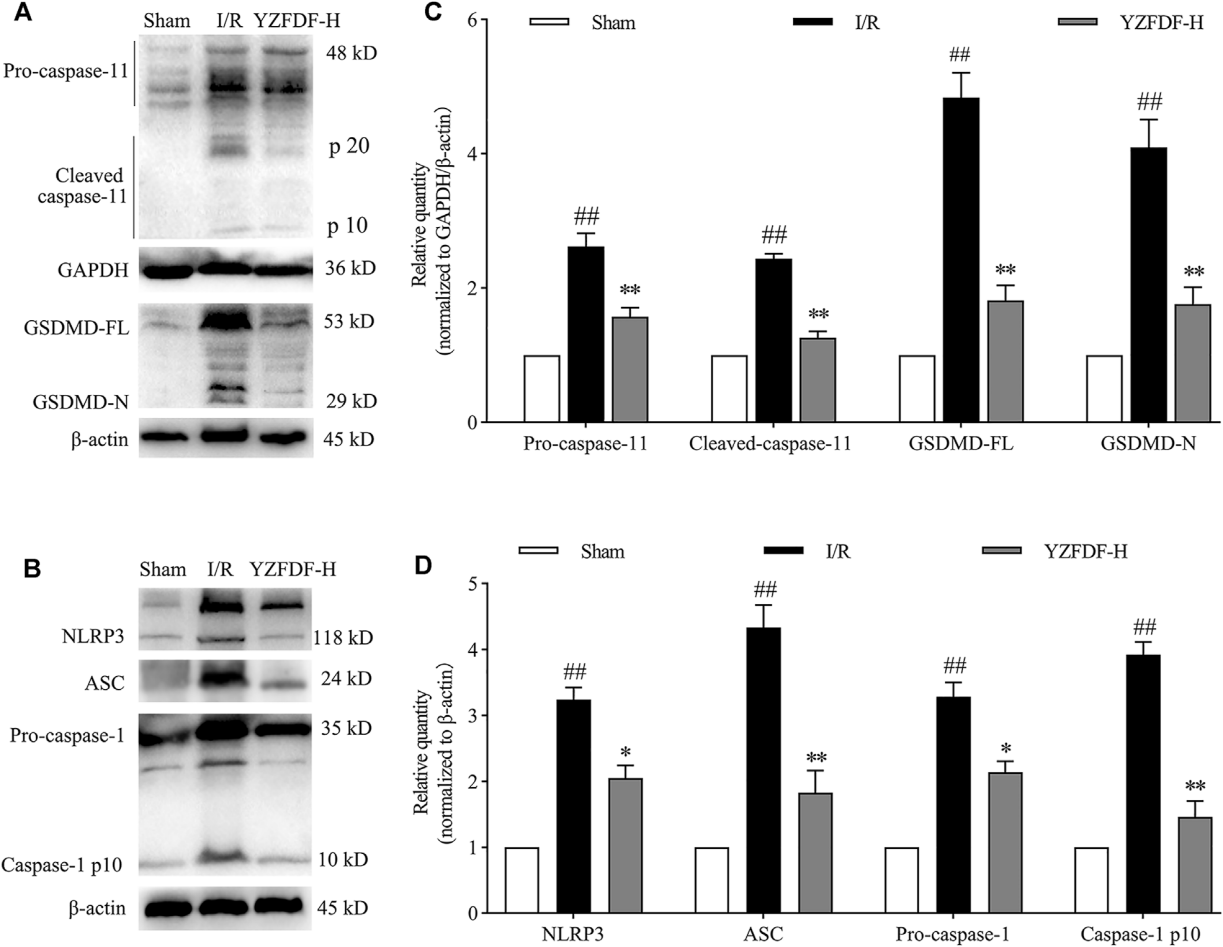
YZFDF inhibited the activation of caspase-11/1 and cleavage of GSDMD at 24 h after cerebral I/R in rats. **(A)** Representative Western blots for pro/cleaved-caspase-11 and GSDMD-FL/N. **(B)** Representative Western blots for NLRP3, ASC, pro-caspase-1, and caspase-1 p10. **(C)** Quantitative analysis of pro/cleaved-caspase-11 and GSDMD-FL/N, *n* = 6. **(D)** Quantitative analysis of NLRP3, ASC, pro-caspase-1, and caspase-1 p10, *n* = 6. ^##^ *p* < 0.01 vs. Sham group; ^*^ *p* < 0.05, ^**^ *p* < 0.01 vs. I/R group.

Our previous study revealed that cerebral I/R activates the caspase-11/GSDMD-mediated pyroptosis pathway. In the present study, the result showed that YZFDF could obviously downregulate the increased protein levels of GSDMD-FL/N (the key effector of pyroptosis) and the related upstream or downstream signaling including pro/cleaved-caspase-11, NLRP3, ASC, and pro/cleaved-caspase-1 after cerebral I/R (Figures 4A–D), indicating that YZFDF could exert inhibitory effects on cerebral I/R–induced pyroptosis.

### Yi-Zhi-Fang-Dai Formula Blocked Overactivation and Pyroptosis of Microglia and Alleviated Inflammatory Responses After Reperfusion

**FIGURE 5:**
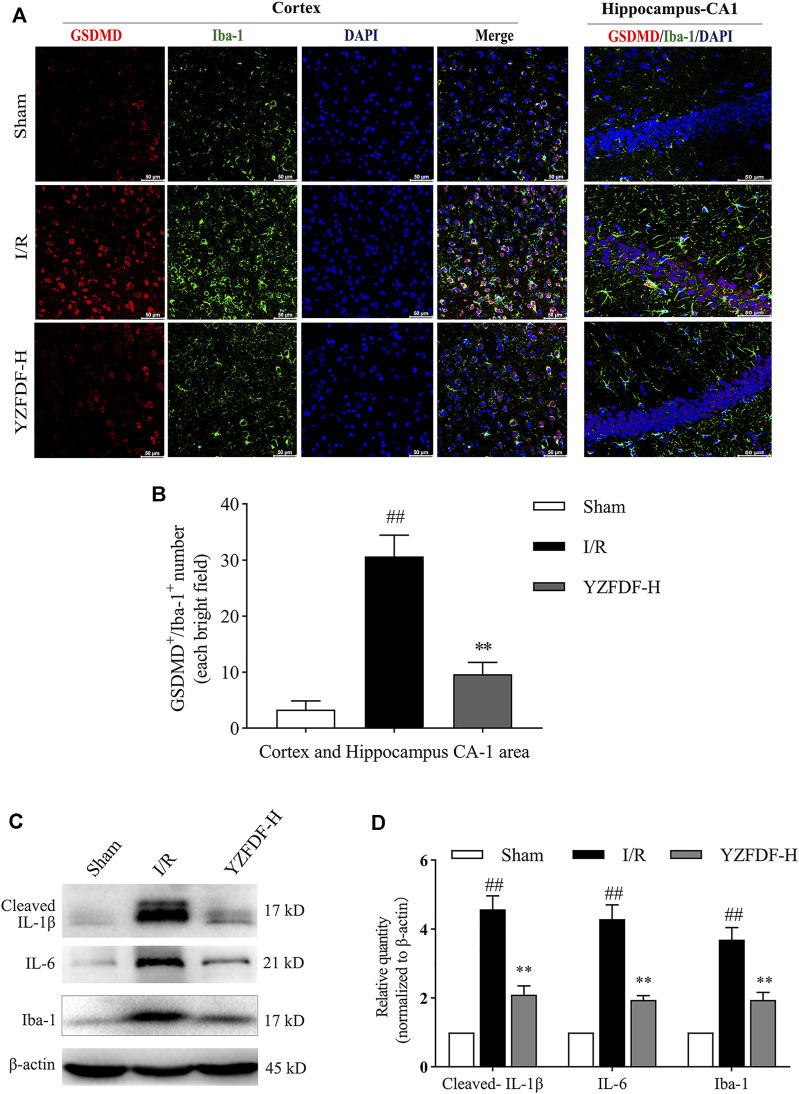
YZFDF blocked overactivation and pyroptosis of microglia and alleviated inflammatory responses at 24 h after cerebral I/R in rats. **(A)** Representative pictures of double immunofluorescence staining of GSDMD (red) colocalized with Iba-1 (green) in cortex and hippocampus-CA1 areas, scale bar = 50 μm. **(B)** Quantitative analysis of double-positive staining of GSDMD^+^/Iba-1^+^ cell number, *n* = 3. **(C)** Representative Western blots for cleaved-IL-1β, IL-6, and Iba-1. **(D)** Quantitative analysis of cleaved-IL-1β, IL-6, and Iba-1, *n* = 6. ^##^ *p* < 0.01 vs. Sham group; ^**^ *p* < 0.01 vs. I/R group.

The inflammatory microenvironment mediated by microglial activation is inherent across the whole course of cerebral I/R injury and deteriorated by pyroptosis after reperfusion, which can be reflected by the expressions of Iba-1 (microglial biomarker), IL-6, and pyroptosis-related pro-inflammatory factors such as IL-1β. The result in the present study showed that YZFDF treatment could reduce the raised immunofluorescent co-staining of GSDMD and Iba-1 in ischemic cortex and hippocampus-CA1 areas (Figures 5A,B) and downregulate the expression levels of Iba-1, IL-6, and cleaved IL-1β after reperfusion (Figures 5C,D), indicating that YZFDF could exert inhibitory effects on inflammatory responses during cerebral I/R by regulating microglial overactivation and pyroptosis.

### Yi-Zhi-Fang-Dai Formula Inhibited Astrocytic Pyroptosis and Protected Against BBB Collapse After Reperfusion

**FIGURE 6:**
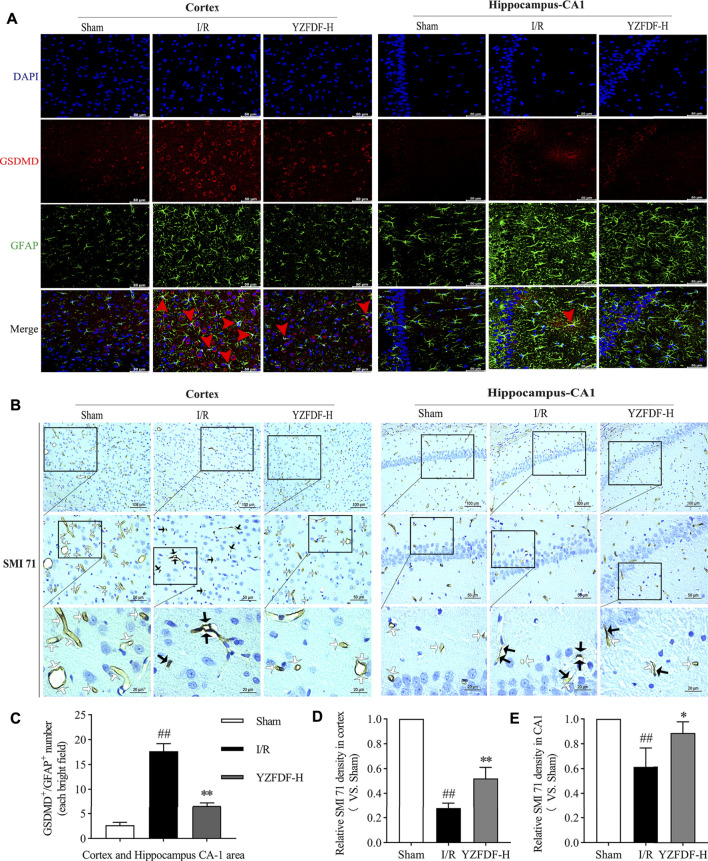
YZFDF inhibited astrocytic pyroptosis and protected against BBB collapse at 24 h after cerebral I/R in rats. **(A)** Representative pictures of double immunofluorescence staining of GSDMD (red) with GFAP (green) in cortex and hippocampus-CA1 areas, scale bars = 50 μm. Red arrows show the GSDMD positive staining localized with the astrocytic nucleus. **(B)** Representative pictures of immunohistochemical staining of SMI 71 (a specific marker for BBB) in cortex and hippocampus-CA1 areas. White arrows show smooth and intact capillaries which represent normal BBB integrity, and black arrows indicate the damage of BBB integrity with unsmoothed, shriveled, or ruptured capillaries, scale bars = 100, 50, and 20 μm, as shown in pictures. **(C)** Quantitative analysis of astrocyte number with GSDMD-positive staining, *n* = 3. **(D)** Quantitative analysis of relative SMI 71 density in the cortex (scale bar = 50 μm), *n* = 3. **(E)** Quantitative analysis of relative SMI 71 density in the hippocampus-CA1 (scale bar = 50 μm), *n* = 3. ^##^ *p* < 0.01 vs. Sham group; ^*^ *p* < 0.05, ^**^ *p* < 0.01 vs. I/R group.

Astrocytic endfeet envelop the cerebral capillaries that form the BBB. Our previous study indicated that astrocytic pyroptosis after I/R is the considerable pathological factor of BBB disruption and shriveled capillaries leading to brain microcirculation disturbance. The result in the present study showed that YZFDF treatment could reduce the GSDMD-positive immunofluorescent staining in astrocytes in ischemic cortex and hippocampus-CA1 areas (Figures 6A,C), indicating that YZFDF could exert inhibitory effects on cerebral I/R–induced astrocytic pyroptosis. Accordingly, YZFDF protected against BBB collapse and reduction after reperfusion which could be observed by immunohistochemical staining of SMI 71 in cortex and hippocampus-CA1 areas of the ischemic hemisphere (Figures 6B,D,E), potentially promoting blood flow circulation in capillaries of ischemic cerebral tissues.

### Yi-Zhi-Fang-Dai Formula Restored AQP-4 Polarization and Reduced Astrocytic Endfeet Swelling and Brain Edema After Reperfusion

**FIGURE 7:**
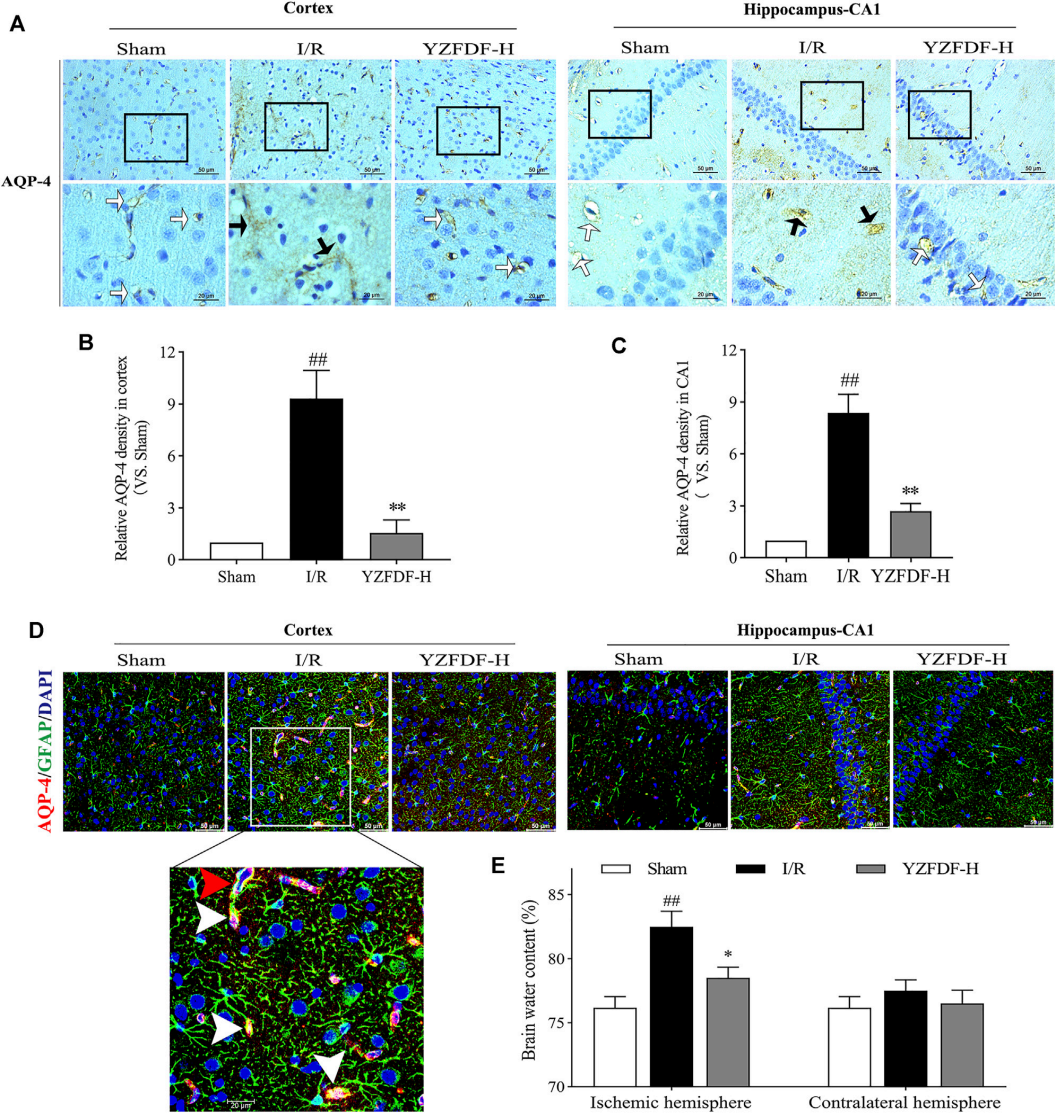
YZFDF restored AQP-4 polarization and reduced astrocytic endfeet swelling and brain edema at 24 h after cerebral I/R in rats. **(A)** Representative pictures of immunohistochemical staining of AQP-4 in cortex and hippocampus-CA1 areas. White straight arrows represent normal AQP-4 polarization, and black arrows represent AQP-4 depolarization with obvious dispersion and perturbed expression, scale bars = 50 and 20 μm, as shown in pictures. **(B)** Quantitative analysis of relative AQP-4 density in the cortex (scale bar = 50 μm), *n* = 3. **(C)** Quantitative analysis of relative AQP-4 density in the hippocampus-CA1 (scale bar = 50 μm), *n* = 3. ^##^ *p* < 0.01 vs. Sham group; ^**^ *p* < 0.01 vs. I/R group. **(D)** Representative pictures of double immunofluorescence staining of AQP-4 (red) with GFAP (green) in cortex and hippocampus-CA1 areas. Red triangle arrow indicates depolarized AQP-4 colocalizes with retracted GFAP and distributes around the astrocytic nucleus, and white triangle arrows indicate depolarized AQP-4 colocalizes with swelling of astrocytic endfeet, scale bars = 50 and 20 μm, as shown in pictures. **(E)** Brain water content analysis of ischemic and contralateral hemispheres, *n* = 4. ^##^ *p* < 0.01 vs. Sham group; ^*^ *p* < 0.05 vs. I/R group.

The loss of AQP-4 polarization on astrocytic endfeet is closely associated with BBB–glymphatic dysfunctions which promote accumulation of metabolites and brain edema. In the present study, our results exhibited the loss of AQP-4 polarization with obvious dispersion, perturbed expressions, and astrocytic endfeet swelling in the ischemic brain tissues, which could be observed by immumohistochemical staining of AQP-4 (Figures 7A–C) and double fluorescence staining of AQP-4 and GFAP in ischemic cortex and hippocampus-CA1 areas (Figure 7D, Supplementary Figure S6), while YZFDF intervention could restore AQP-4 polarization and accordingly reduce astrocytic endfeet swelling and brain edema in the ischemic hemisphere after reperfusion (Figures 7A–E).

### Yi-Zhi-Fang-Dai Formula Promoted Aβ Clearance and Prevented the Formation of Aβ_1-42_ Oligomers After Reperfusion

**FIGURE 8:**
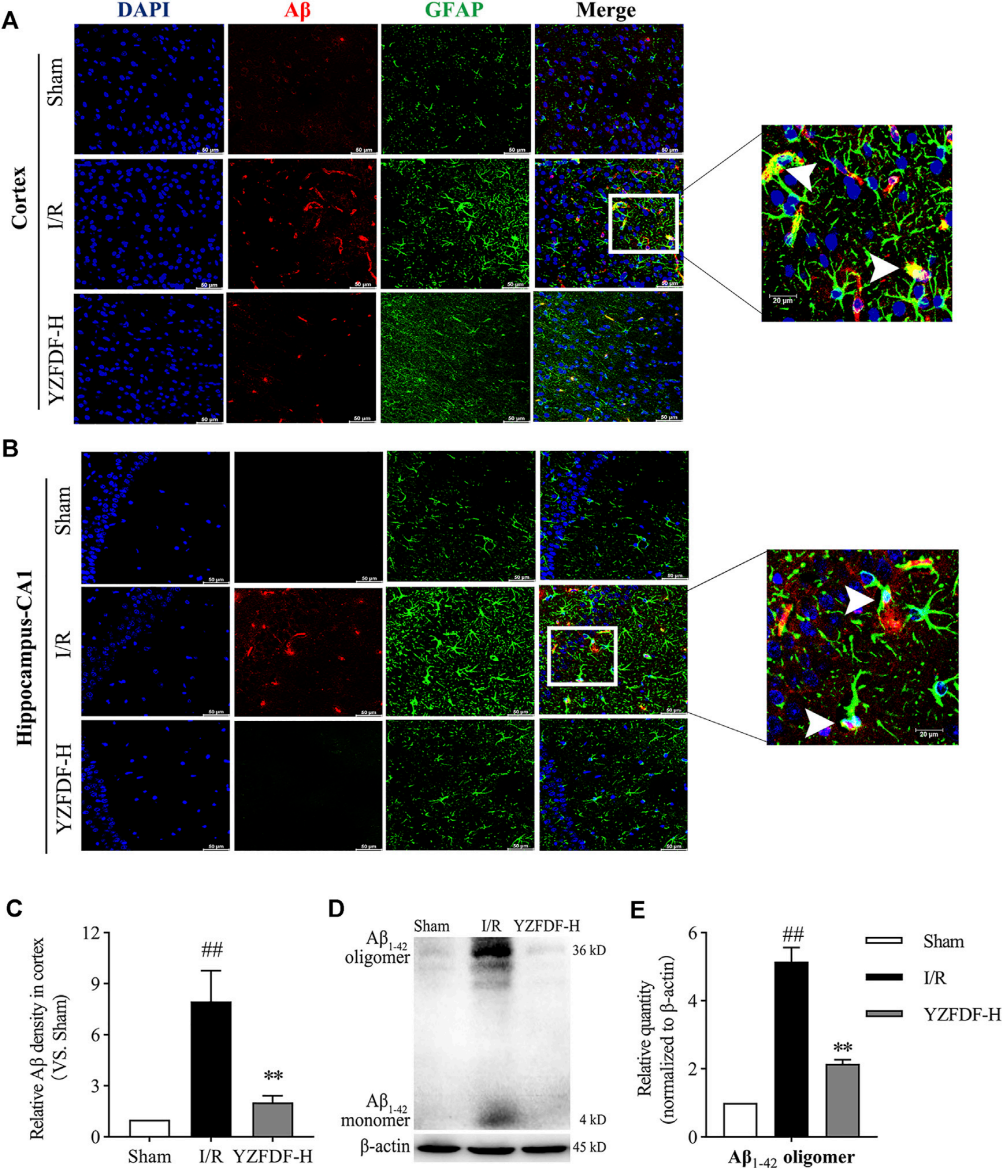
YZFDF promoted Aβ clearance and prevented the formation of Aβ_1-42_ oligomers at 24 h after cerebral I/R in rats. **(A,B)** Representative pictures of double immunofluorescence staining of Aβ (red) with GFAP (green) in cortex and hippocampus-CA1 areas, and white arrows represent Aβ accumulation in sites of swelling astrocytic endfeet, scale bars = 50 and 20 μm, as shown in pictures. **(C)** Quantitative analysis of relative Aβ density in the cortex (scale bar = 50 μm), *n* = 3. **(D)** Representative Western blots for the Aβ_1-42_ monomer and Aβ_1-42_ oligomer. **(E)** Quantitative analysis of Aβ_1-42_ oligomers, *n* = 6. ^##^ *p* < 0.01 vs. Sham group; ^**^ *p* < 0.01 vs. I/R group.

Based on the above results in the present study, our study further exhibited that Aβ accumulates in the sites of swelling astrocytic endfeet, which was consistent with the situation of AQP-4 polarization loss (Figures 8A–C). However, YZFDF treatment could alleviate Aβ acute accumulation around astrocytes within 24 h after reperfusion in ischemic cortex and hippocampus-CA1 areas (Figures 8A–C). Furthermore, YZFDF could prevent the formation of Aβ_1-42_ oligomers (the main form of toxic Aβ) in ischemic brain tissues after reperfusion (Figures 8D,E).

### Yi-Zhi-Fang-Dai Formula Alleviated Neuronal Damage and Promoted Neuron Survival After Reperfusion

**FIGURE 9:**
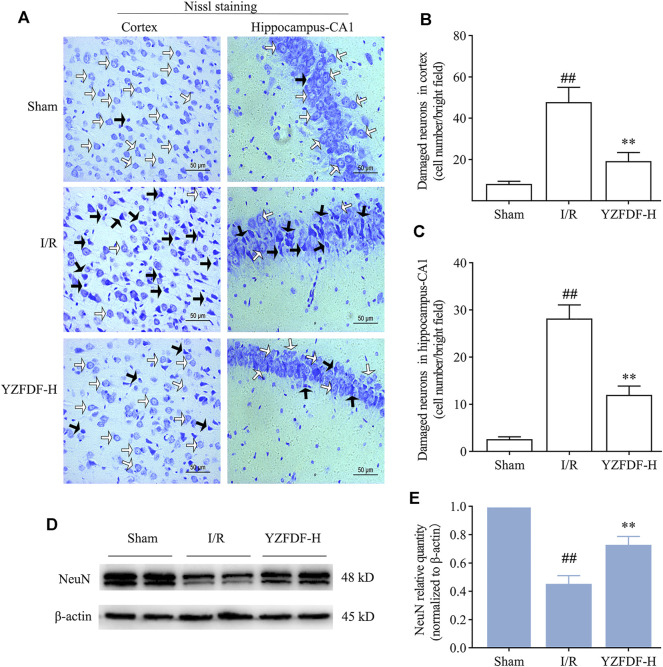
YZFDF alleviated neuronal damage and promoted neuron survival at 24 h after cerebral I/R in rats. **(A)** Representative pictures of Nissl staining in ischemic cortex and hippocampus-CA1 areas. White arrows represent normal morphology of neurons with the clear nucleolus, abundant cytoplasm, and intact structure, and black arrows represent damaged neurons appearing shrunken and deep stained, scale bars = 50 μm. **(B,C)** Quantitative analysis of damaged neurons in cortex and hippocampus-CA1 areas, *n* = 4. ^##^ *p* < 0.01 vs. Sham group; ^**^ *p* < 0.01 vs. I/R group. **(D)** Representative Western blots for NeuN. **(E)** Quantitative analysis of NeuN, *n* = 6. ^##^ *p* < 0.01 vs. Sham group; ^**^ *p* < 0.01 vs. I/R group.

The above results in the present study have revealed that YZFDF could inhibit cerebral I/R–induced pyroptosis, damage of the BBB–glymphatic system, and Aβ accumulation after reperfusion. Accordingly, our study further showed that YZFDF could alleviate neuronal damage after reperfusion (Figures 9A–C) and promote neuron survival in ischemic cortex and hippocampus-CA1 areas (Figures 9D,E), which was consistent with the results of the neurological function assessment and measurement of the cerebral infarct area.

## Discussion

AD is a chronic cerebral disease affected by multifaceted etiological factors, among which cerebrovascular diseases represented by ischemic stroke have been attracting great attention (Wang R. et al., 2021; Eskandari et al., 2021; Zupanic et al., 2021). Despite the apparent association between AD and ischemic stroke, it remains unclear how the latter induces the onset and development of AD. However, accumulated evidence has showed that the neuroinflammation-related dysfunctional BBB–glymphatic system is responsible for triggering Aβ accumulation in the brain and may represent key link between ischemic stroke and dementia (Goulay et al., 2020; Ronaldson and Davis, 2020).

Restoring blood flow of the ischemic cerebral tissue in a short-time window is the most important therapeutic measure for patients suffering from acute cerebral ischemia. However, the potential additional injury following ischemia/reperfusion (I/R) has a great impact on the therapeutic efficacy of restoring blood flow (Kalogeris et al., 2016). I/R injury is the common pathophysiological phenomenon liable to occur in multiple organs including brain, and deterioration of microvasculature damage after reperfusion and the following non-reflow phenomenon of capillaries is the basic pathological change of I/R injury (Kloner et al., 2018). In the brain, the BBB is the main structure of microvasculature, and microcirculation disturbance resulting from BBB breakdown plays a crucial role in cerebral I/R injury (Mohamed Mokhtarudin and Payne, 2015; Huang et al., 2020; Xiao et al., 2020). Furthermore, BBB breakdown is recognized as an early marker for the onset of AD (Bell and Zlokovic, 2009; Sagare et al., 2012; Nation et al., 2019). Therefore, BBB protection during cerebral I/R injury is considered an important strategy for the prevention and treatment of ischemic stroke and poststroke dementia (Gursoy-Ozdemir et al., 2012; Goulay et al., 2020; Ronaldson and Davis, 2020).

As for the medication of cerebral I/R injury and BBB protection, amounts of literature indicated that various extracts or compound medicines from natural products show beneficial effects (Li Y. et al., 2019; Yu et al., 2020). For instance, Buyang Huanwu decoction (BHD) and Tongxinluo (TXL), both consisting of herbal and animal medicines, are common TCM prescriptions used for treating Qi deficiency and blood stasis syndrome of ischemic stroke by efficacies of invigorating Qi and activating blood circulation (Su et al., 2011; Wang Y. et al., 2020). Experimental studies revealed that BHD and TXL could alleviate cerebral I/R injury and exert protective effects on the BBB (Liu et al., 2013; Chen et al., 2019). In studies about the therapeutic efficacy of TCM compound medicines or extracts, such as BHD and TXL mentioned above for cerebral I/R injury, drug administration for 3–7 days starting before MCAO is the common means to enhance intervention effects (Liu et al., 2013; Yu et al., 2018; Chen et al., 2019; She et al., 2019; Zheng et al., 2019; Zhang S. et al., 2021). Accordingly, based on these literatures, we select YZFDF pretreatment to carry out the present study. However, different from BHD and TXL, YZFDF is purely composed of several plant-derived natural products, but the preliminary results in the present study showed that YZFDF could also exert significant neuroprotective effects against cerebral I/R injury by dose-dependently alleviating neurological deficits and cerebral infarct after reperfusion. Our previous work revealed that pyroptosis of glial cells (microglia and astrocytes) is a considerable pathological mechanism causing BBB damage after cerebral I/R, and abating pyroptosis contributes to protect against BBB breakdown and maintains the homeostasis of brain microenvironments (Lyu et al., 2021). Our present study further indicated that YZFDF could protect ischemic cerebral tissues against pyroptotic cell death and accordingly exert protective effects against BBB collapse in damaged cortex and hippocampus areas after cerebral I/R, potentially promoting blood flow reperfusion in microcirculation of ischemic cerebral tissues, which probably owed to the multiple efficacies of invigorating Qi, removing blood stasis, and dredging brain collaterals.

As pro-inflammatory programmed cell death is distinguished from apoptosis and necrosis, pyroptosis manifests as nanopore formation on the cytomembrane leading to cell swelling and death, which is executed by the N-terminal fragments of gasdermin family represented by GSDMD-N (Feng et al., 2018). Upon stimulation, NLRP3 recruits pro-caspase-1 through the adapter molecule ASC to form the NLRP3/ASC/caspase-1 inflammasome, resulting in the activation of caspase-1 to cleave GSDMD and then leads to the secretion of pro-inflammatory cytokines, which is called canonical pyroptosis (Patel et al., 2017; Cong et al., 2020). However, in the caspase-11/GSDMD-mediated noncanonical pyroptosis pathway, GSDMD-N formed by the activation of caspase-11 acts as the upstream signaling that activates the NLRP3/ASC/caspase-1 inflammasome to further cause the maturation and secretion of pro-inflammatory cytokines such as IL-1β (Kayagaki et al., 2015; Aglietti et al., 2016). Caspase-11 is previously considered to be activated by lipopolysaccharide in infectious diseases (Hagar et al., 2013). Cerebral I/R injury is a noninfectious pathological process (Dong et al., 2018), and thus previous studies attributed cerebral I/R–induced pyroptosis to its canonical pathway (She et al., 2019). However, emerging literature has proven that caspase-11/GSDMD-mediated noncanonical pyroptosis is involved in acute kidney injury and hepatic injury induced by some endogenous pathophysiological factors including I/R injury (Miao et al., 2019; Wang X. et al., 2020). Our recent work revealed that caspase-11/GSDMD-mediated noncanonical pyroptosis also involves in cerebral I/R injury (Lyu et al., 2021). In the present study, we observed that YZFDF could obviously inactivate caspase-11 as well as cut off NLRP3/ASC/caspase-1 signaling and thus inhibit the cleavage of GSDMD to reduce the formation of GSDMD-N, indicating that YZFDF could exert inhibitory effects on cerebral I/R–induced canonical and noncanonical pyroptosis.

BBB breakdown in the cerebral ischemic period mainly results from the interaction between blood components (activated leukocytes and platelets) and microvascular endothelial cells, leading to inflammatory response and microthrombosis, which are exacerbated by blood flow reperfusion and then followed by the no-reflow phenomenon (Kalogeris et al., 2016). Following the initial ischemic damage, a wave of detrimental secondary events is caused by reperfusion such as oxidative stress and acute inflammation. Because inflammatory response is inherent across the whole course of cerebral I/R injury, and additionally due to the exacerbation of thromboinflammation and no-reflow after reperfusion in capillaries, neuroinflammation is recognized as the most vital pathological factor impacting on the BBB and the fundamental therapeutic target during both ischemic period and reperfusion (Liu et al., 2014; Li WH. et al., 2019; Stoll and Nieswandt, 2019). The results in our present study showed that YZFDF pretreatment could obviously downregulate the levels of IL-6 and inhibit the aberrant activation of microglia, reflecting that YZFDF alleviated the acute neuroinflammation during the course of cerebral I/R. There are intimate relationships between inflammation and pyroptosis (Kesavardhana et al., 2020). Research has proven that infectious or sterile inflammation can stimulate occurrence of pyroptosis, while pyroptosis reversely aggravates inflammatory responses by further generating and releasing certain pro-inflammatory mediators (Patel et al., 2017). In present study, our results further indicated that YZFDF could exert obvious inhibitory effects on microglial pyroptosis and the generation of IL-1β, which provided evidence for the blocking effect of YZFDF on cross talks between neuroinflammation and pyroptosis after reperfusion.

In addition to microglia, astrocytes are as well the main locations of cerebral I/R–induced pyroptosis which peaks at 24 h after reperfusion (Zhang et al., 2019; Lyu et al., 2021). As the most abundant glial cells in the mammalian brain, astrocytes are the major supporter of energy supply and nutrition for neurons in neuro-glial-vascular coupling (Nortley and Attwell, 2017; Allen and Lyons, 2018), and moreover astrocytic endfeet function as essential components of both the BBB and glymphatic system to clear metabolites such as Aβ and maintain brain microenvironmental homeostasis, in which aquaporin-4 (AQP-4) on astrocytic endfeet plays an important role (Gleiser et al., 2016; Rasmussen et al., 2021). Thus, in our previous study (Lyu et al., 2021), astrocytic pyroptosis was considered a vital factor causing the BBB further disruption and AQP-4 polarization loss, which account for Aβ accumulation, brain edema formation, non-reflow phenomenon of capillaries, and neuronal damage after cerebral I/R. In the present study, our results showed that YZFDF could obviously inhibit astrocytic pyroptosis after reperfusion which contributed to restore AQP-4 polarization and reduce brain edema. Moreover, the results as well showed that based on the protective effects against BBB–glymphatic dysfunctions, YZFDF could further significantly promote Aβ clearance and prevented the formation of Aβ_1-42_ oligomers after reperfusion.

Aβ accumulation is the essential factor in the biological definition of AD, which can further cause tau pathology and neuron loss (Jack et al., 2018). Furthermore, Aβ accumulation in the brain can lead to extensive damages of the neurovascular unit (NVU) which comprises neurons, perivascular microglia, and BBB including cerebral microvascular endothelial cells (CMECs), pericytes, and surrounding astrocytes (Yamazaki and Kanekiyo, 2017). As mentioned previously, accumulated Aβ can act on pericytes via evoking reactive oxygen species generation in the form of oligomers to constrict capillaries which further promotes energy lack of neurons and neurodegeneration (Nortley et al., 2019). Aβ accumulation in capillaries, associated with cerebral amyloid angiopathy (CAA), also affect NVU astrocytes to cause mislocalization of AQP-4 expression (Wilcock et al., 2009), which was consistent with the acute accumulation of Aβ around swelling astrocytic endfeet as shown in our present study. Our previous study indicated that Aβ_1-42_ oligomers are the main form of toxic Aβ and that Aβ_1-42_ oligomers could damage tight junction scaffold proteins among CMECs to induce BBB leakage via the receptor for advanced glycation end product (RAGE) (Wan W. et al., 2014; Wan et al., 2015; Chan et al., 2018; Chen et al., 2018). Previous studies demonstrated that Aβ increasingly accumulates around astrocytes along with both BBB breakdown and delayed neuronal death in the hippocampus within 6 months after cerebral I/R and even deposits as plaques with time further extension (van Groen et al., 2005; Pluta et al., 2010), which provides experimental evidence for stroke-inducing sporadic AD. Furthermore, recently, Martins et al. revealed that Aβ oligomers potentially resulting from activated platelets in microthrombosis massively accumulate in brain tissues including capillaries within 24 h after cerebral I/R, and they further demonstrated that Aβ oligomers are responsible for some of the brain damage during stroke by the property of forming ion channels on the cytomembrane (in a non-receptor–dependent way) to affect cellular osmotic balance and promote brain edema formation (Martins et al., 2019). Our recent study revealed that pyroptosis accounts for dysfunctions of the BBB–glymphatic system and the acute accumulation of toxic Aβ within 24 h after cerebral I/R (Lyu et al., 2021). However, on the other hand, toxic Aβ has been identified as a cause of pyroptosis and neuroinflammation in previous studies (Halle et al., 2008; Shi F. et al., 2015), which suggests a magnified effect of Aβ accumulation after cerebral I/R.

Large amounts of evidence mentioned previously indicate that Aβ accumulation is not only the consequence but also the further cause of the dysfunctional BBB–glymphatic system and neuronal damage in the course of cerebral I/R injury and in the process of ischemic stroke–inducing dementia. Therefore, maintaining Aβ clearance and protecting brain tissues against Aβ toxicity after cerebral I/R could offer a new viewpoint to alleviate ischemic stroke and prevent poststroke dementia. TCM herbal formulas or extracts have shown unique advantages in the prevention and treatment of complex brain diseases including acute ischemic stroke and dementia (Li Y. et al., 2019; Yu et al., 2020). Our previous study demonstrated that YZFDF could exert protective effects against Aβ_1-42_ oligomer–induced BBB and neuronal damages (Liu et al., 2016a; Chan et al., 2020). The present study showed that YZFDF could promote Aβ clearance to prevent Aβ acute accumulation and the formation of Aβ_1-42_ oligomers and thus block the potential interaction between the BBB–glymphatic dysfunctions and Aβ accumulation. Viewed from the macro perspective of TCM holism, the four herbal medicines of YZFDF potentially possess the neuroprotective effects against cerebral I/R injury by synergistically exerting efficacies of invigorating Qi, removing blood stasis, and dredging brain collaterals based on their drug properties. From the modern pharmacological and microcosmic perspective, our previous study had identified the bioactive ingredients in YZFDF, which contain bilobalide and ginkgolide A of *Ginkgo biloba* leaves, ginsenoside Rg1 of ginseng, cistanoside A of Cistanches Herba, and α-asarone of grassleaf sweetflag, which have a wide range of activities including anti-inflammation, anti-aggregation of platelets and proteins, neurovascular protections, and neurotrophic effects against cerebral I/R injury (Kimura et al., 1988; Zhou et al., 2014; Liu et al., 2016a; Zheng et al., 2019; Sarkar et al., 2020; Zhang K. et al., 2021). Our previous studies indicated that EGb761, a product extracted from *Ginkgo biloba* leaves (the main herb of YZFDF), could regulate Aβ-induced microglial inflammatory responses, BBB disruption, and alleviate neuronal damage (Wan WB. et al., 2014; Liu et al., 2016b; Wan et al., 2016). As a consequence, in this study, YZFDF potentially exerted advantages of multiple bioactive ingredients, multiple effects, and multiple targets on the whole NVU to alleviate cerebral I/R–induced neuroinflammation, pyroptosis, BBB–glymphatic dysfunctions, Aβ accumulation, and their interactions. Nevertheless, the action on body systems of YZFDF in this study and post-treatment and long-term effects as well as the more detailed targets and mechanism remain to be clarified and deserve our further explorations in future research.

## Conclusion

In summary, we demonstrated the neuroprotective properties of YZFDF against cerebral I/R injury at the first stage, and the following study further indicated that YZFDF pretreatment could exert inhibitory effects on microglial and astrocytic pyroptosis and acute neuroinflammation, which fundamentally contribute to restore the BBB–glymphatic functions, promote Aβ clearance and prevent the formation of Aβ oligomers via protecting against BBB breakdown, and AQP-4 polarization loss and thus facilitates to maintain the homeostasis of brain microenvironments and neuron survival after cerebral I/R.

## Data Availability

The original contributions presented in the study are included in the article/Supplementary Material, further inquiries can be directed to the corresponding authors.
