# Supplementary figures and images for: Yi-Zhi-Fang-Dai Formula Exerts Neuroprotective Effects Against Pyroptosis and Blood–Brain Barrier–Glymphatic Dysfunctions to Prevent Amyloid-Beta Acute Accumulation After Cerebral Ischemia and Reperfusion in Rats
Source: Front Pharmacol. 2021 Dec 15;12:791059. doi: 10.3389/fphar.2021.791059 (PMC8714930; doi:10.3389/fphar.2021.791059)

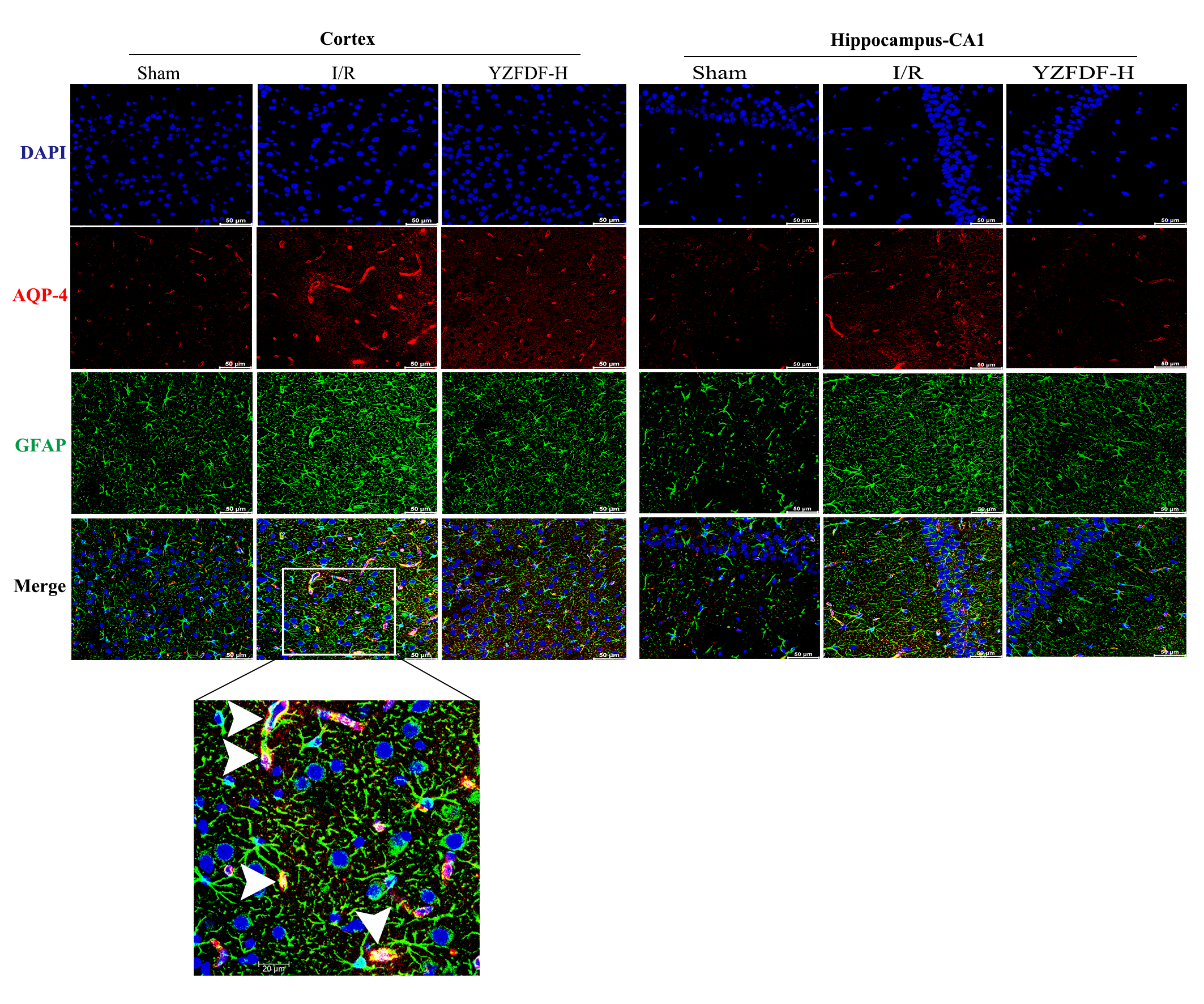

Supplement: Supplementary file 1 [file Image6.TIF]

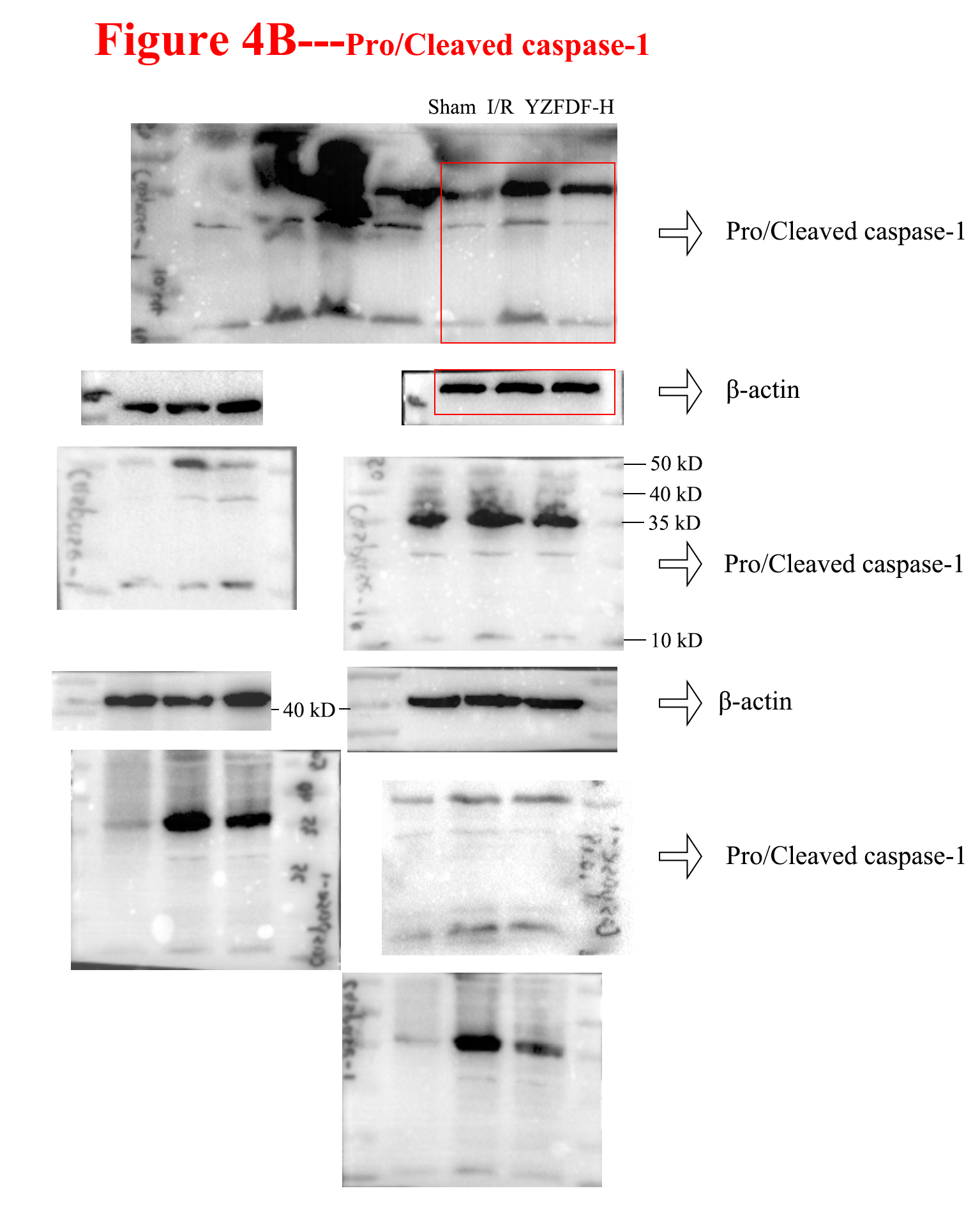

Supplement: Supplementary file 2 [file Image3.TIF]

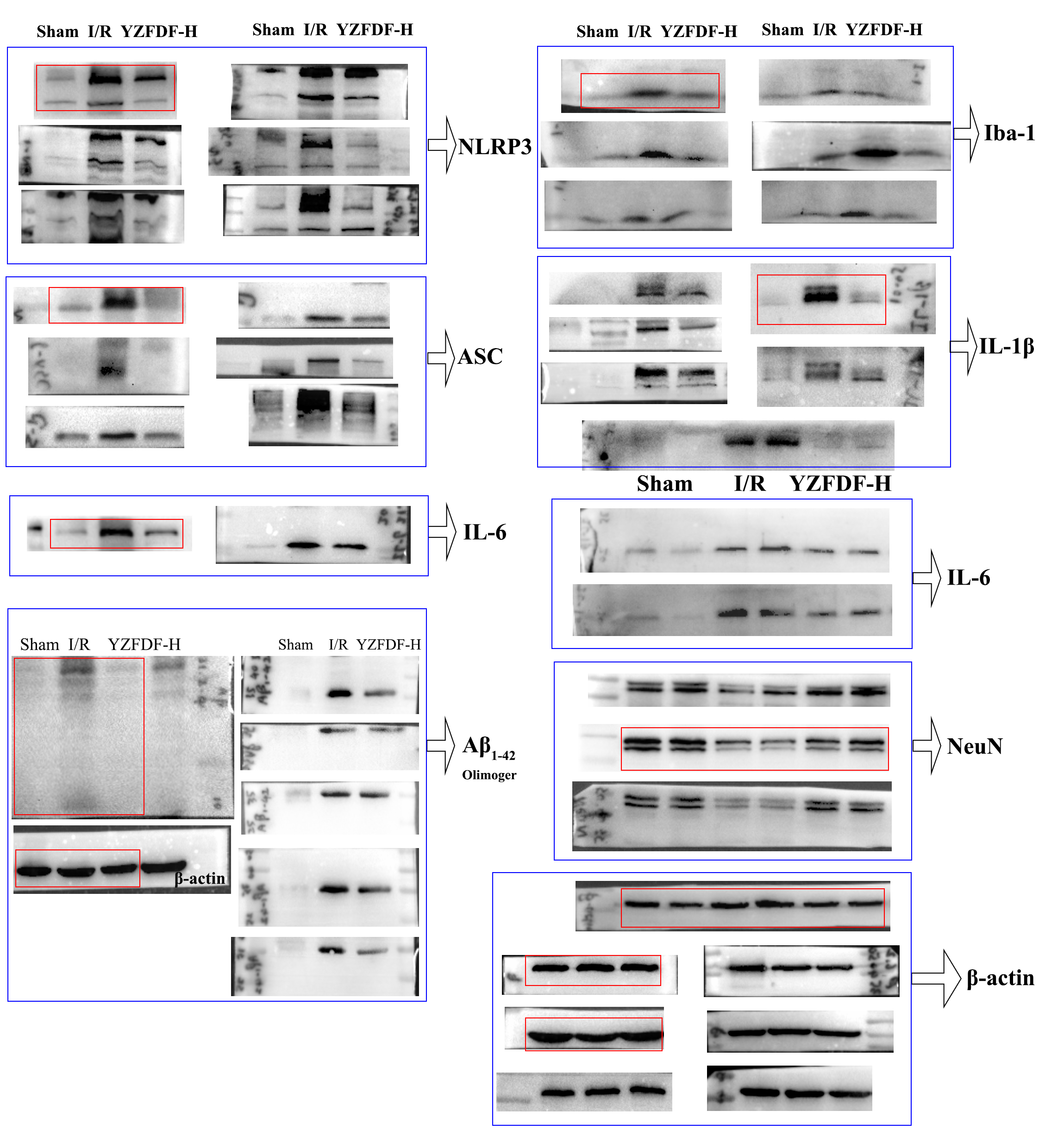

Supplement: Supplementary file 3 [file Image4.TIF]

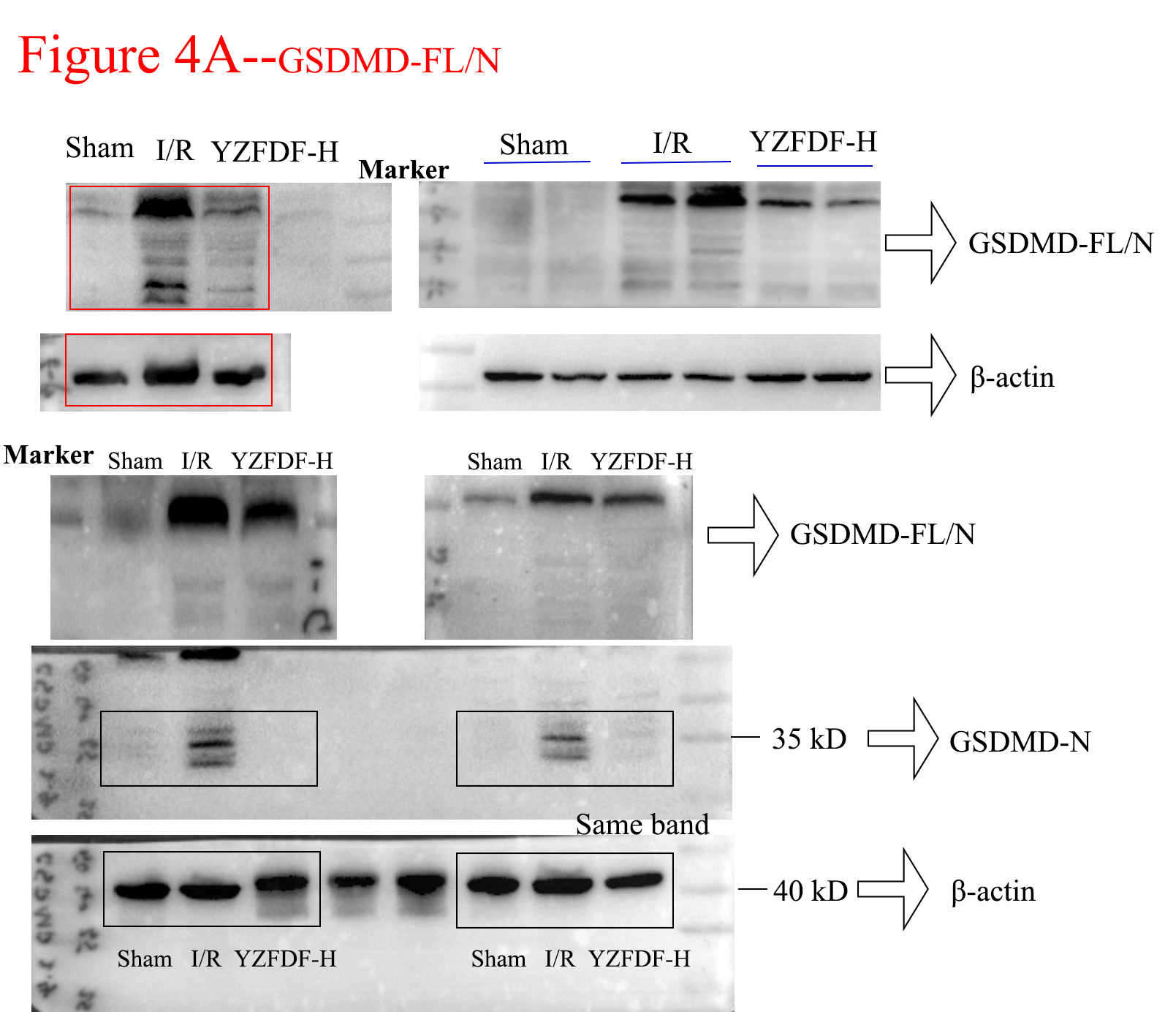

Supplement: Supplementary file 4 [file Image2.TIF]

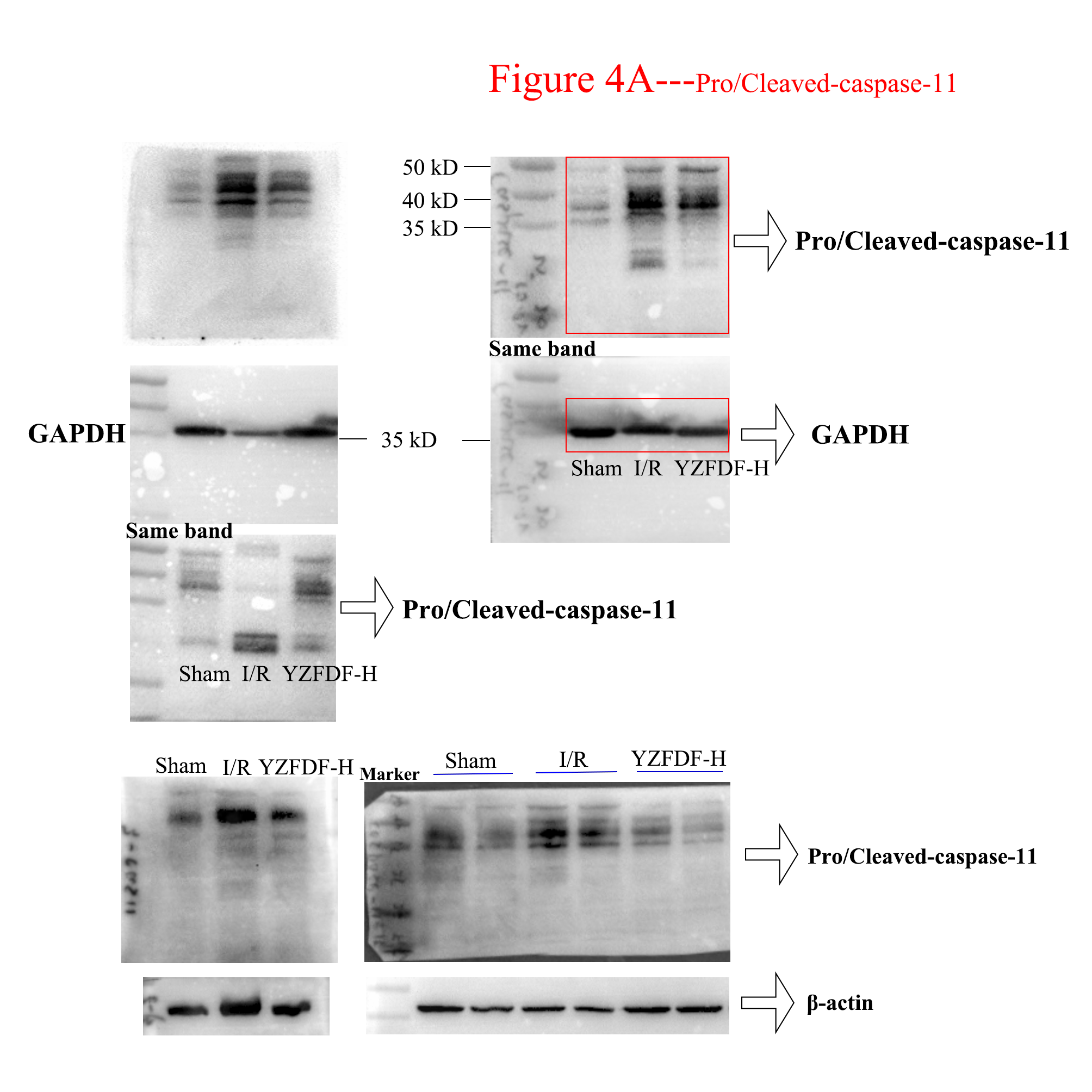

Supplement: Supplementary file 5 [file Image1.TIF]

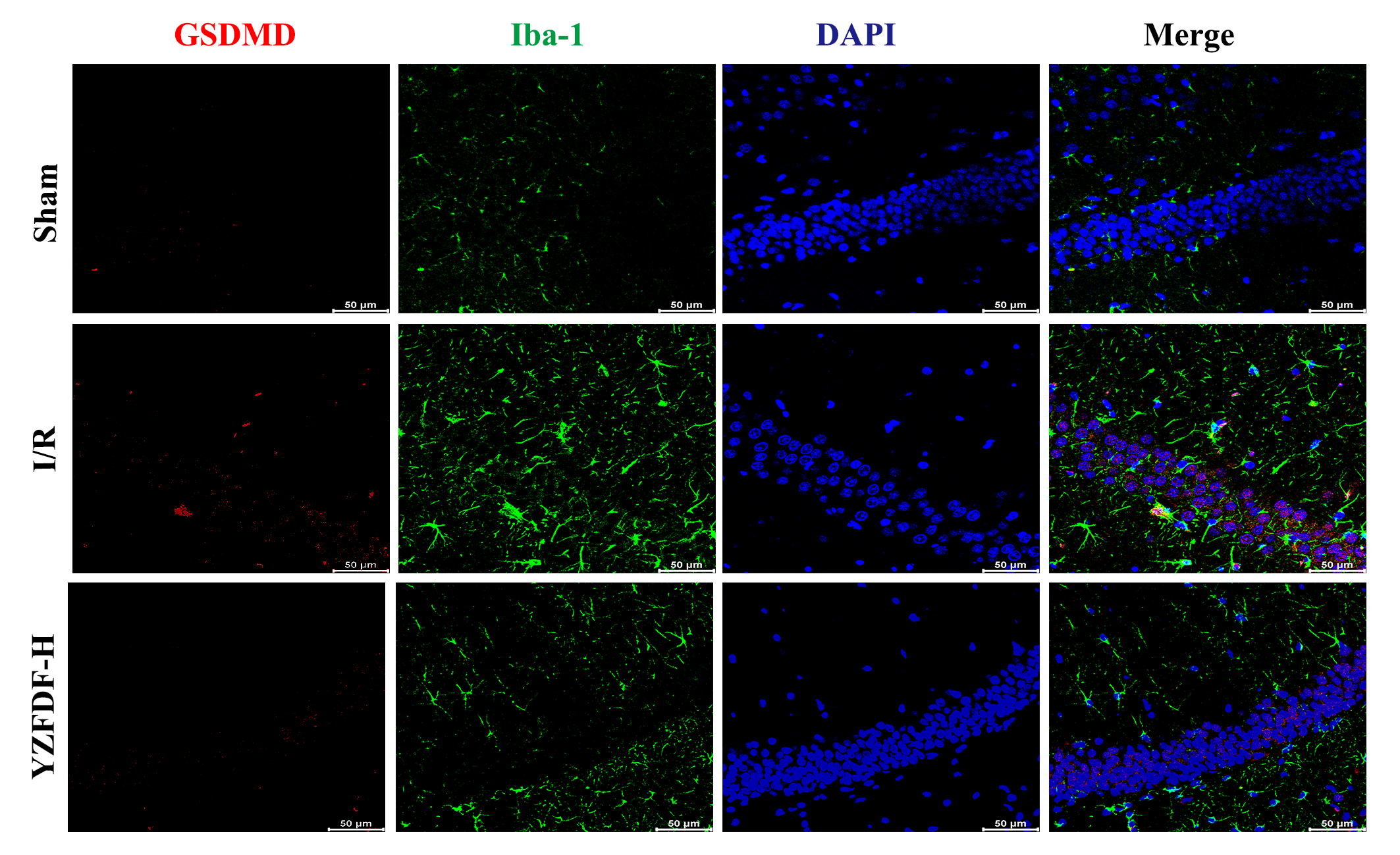

Supplement: Supplementary file 7 [file Image5.TIF]
