# Supplementary material for: Yi-Zhi-Fang-Dai Formula Exerts Neuroprotective Effects Against Pyroptosis and Blood–Brain Barrier–Glymphatic Dysfunctions to Prevent Amyloid-Beta Acute Accumulation After Cerebral Ischemia and Reperfusion in Rats
Source: Front Pharmacol. 2021 Dec 15;12:791059. doi: 10.3389/fphar.2021.791059 (PMC8714930; doi:10.3389/fphar.2021.791059)
Supplement: Supplementary file 6 [file Table1.DOC]

**TABLE S1** Detailed information of primary antibodies employed in this study

| **Antibody** | **Type** | **Dilution** | **Source** | **Cat. No.** |
| --- | --- | --- | --- | --- |
| GSDMD | Rabbit polyclonal | 1:1000, WB | CST | 93709 |
| GSDMD | Rabbit polyclonal | 1:100, IF | Proteintech | 20770-1-AP |
| GSDMD | Mouse monoclonal | 1:200, IF | Santa Cruz | sc-393581 |
| caspase-11 | Rat monoclonal | 1:200, WB | Santa Cruz | sc-56038 |
| NLRP3 | Rabbit polyclonal | 1:300, WB | Proteintech | 19771-1-AP |
| ASC | Mouse monoclonal | 1:200, WB | Santa Cruz | sc-514414 |
| caspase-1 | Rabbit polyclonal | 1:500, WB | Proteintech | 22915-1-AP |
| IL-6 | Mouse monoclonal | 1:200, WB | Santa Cruz | sc-57315 |
| IL-1β | Armenian hamster monoclonal | 1:200, WB | Santa Cruz | sc-12742 |
| Iba-1 | Rabbit monoclonal | 1:1000, WB | Abcam | Ab178847 |
| Iba-1 | Rabbit polyclonal | 1:100, IF | Proteintech | 10904-1-AP |
| GFAP | Rabbit polyclonal | 1:100, IF | Proteintech | 23935-1-AP |
| GFAP | Mouse monoclonal | 1:100, IF | Proteintech | 60190-1-Ig |
| NeuN | Rabbit polyclonal | 1:1000, WB | Proteintech | 26975-1-AP |
| SMI 71 | Mouse monoclonal | 1:100, IHC | Biolegend | 836812 |
| AQP-4 | Mouse monoclonal | 1:100, IF; 1:200, IHC | Santa Cruz | sc-32739 |
| Aβ | Mouse monoclonal | 1:200, IF | Santa Cruz | sc-28365 |
| Aβ_1-42_ | Rabbit monoclonal | 1:1000, WB | Abcam | ab201060 |
| β-actin | Mouse monoclonal | 1:1000, WB | CST | 3700 |
